# The Role of Zinc in Selected Female Reproductive System Disorders

**DOI:** 10.3390/nu12082464

**Published:** 2020-08-16

**Authors:** Marzenna Nasiadek, Joanna Stragierowicz, Michał Klimczak, Anna Kilanowicz

**Affiliations:** Department of Toxicology, Medical University of Lodz, Muszyńskiego 1, 90-151 Lodz, Poland; joanna.stragierowicz@umed.lodz.pl (J.S.); michal.klimczak@umed.lodz.pl (M.K.)

**Keywords:** zinc, supplementation, ovary, uterus, reproductive system, polycystic ovary syndrome (PCOS), endometriosis, dysmenorrhea, primary dysmenorrhea (PD)

## Abstract

Zinc is an essential microelement that plays many important functions in the body. It is crucial for the regulation of cell growth, hormone release, immunological response and reproduction. This review focuses on its importance in the reproductive system of women of reproductive and postmenopausal ages, not including its well described role in pregnancy. Only recently, attention has been drawn to the potential role of zinc in polycystic ovary syndrome (PCOS), dysmenorrhea, or endometriosis. This review is mainly based on 36 randomized, controlled studies on reproductive, pre- and post-menopausal populations of women and on research trying to explain the potential impact of zinc and its supplementation in the etiology of selected female reproductive system disorders. In women with PCOS, zinc supplementation has a positive effect on many parameters, especially those related to insulin resistance and lipid balance. In primary dysmenorrhea, zinc supplementation before and during each menstrual cycle seems to be an important factor reducing the intensity of menstrual pain. On the other hand, little is known of the role of zinc in endometriosis and in postmenopausal women. Therefore, further studies explaining the potential impact of zinc and its supplementation on female reproductive system would be highly advisable and valuable.

## 1. Introduction

### 1.1. General Information

Zinc (Zn) is an essential microelement that is present in all body tissues and fluids, mainly intracellularly. The total amount of zinc in the human body is estimated at 2–3 g and less than 0.2% of it is found in plasma, where its concentration is about 15 µmol/L (100 µg/dL) [1,2,3,4,5]. Approximately 0.1% of the zinc content of the body (i.e., 2–3 mg) needs to be supplied daily [6,7]. The zinc status in humans depends on gender, age, physiological condition and diet. Most international groups (e.g., World Health Organization/Food and Agriculture Organization (WHO/FAO), Institute of Medicine (IOM) or European Food Safety Authority (EFSA)) have developed dietary recommendations for zinc [8,9,10], which are presented in Table 1.

Many different foods contain zinc, but that of animal-origin (organs and flesh of mammals, fish, eggs and dairy products) are the richest source of well absorbable zinc. Plant-based foods, such as cereals, grains, nuts and legumes contain smaller and less efficiently absorbed amounts of this element [1,3,10,11,12]. Zinc bioavailability depends on the chemical form on zinc, its solubility and the presence of other substances in the food influencing the efficiency of zinc absorption [11]. Generally, its absorption increases with protein intake. Animal proteins improve the bioavailability of zinc from plant food sources (by counteracting the inhibitory effect of phytates) [10,12]. Soluble ligands or chelators of zinc (e.g., EDTA, amino acids, organic acids) have a positive effect on its absorption by increasing zinc solubility [12]. The main inhibitor of this process is phytate (myoinositol hexaphoshate—P), which is present in many plant foods and irreversibly binds zinc in the intestinal lumen disturbing its absorption [11]. Cereals (e.g., white rice—the dietary basis in many Asian countries) and legumes (e.g., bean, which is very popular in Latin America) contain the most phytate. To estimate the likely absorption of zinc from the diet, the P:Zn molar ratio can be applied. It is generally believed that diets with a P:Zn molar ratio >15 have relatively poor zinc bioavailability (10–15%, “low-bioavailability diet”), those with a P:Zn molar ratio between 5 and 15 have medium zinc bioavailability (30–35%, “medium-bioavailability diet”), and those with a P:Zn molar ratio <5 have relatively good zinc bioavailability (45–55%, “high-bioavailability diet”) [11,12,13].

The main cause of Zn deficiency is inadequate dietary intake, which is common in many parts of the world [12]. According to the data from 2001, almost half of the global population at that time was at risk of zinc deficiency. Although the zinc intake from diet is on average 10 mg Zn/day, around 1.5 mg of zinc is absorbable, mainly due to the phytate content in the diet [11]. Despite the fact that most people in developed countries are on a medium-bioavailability diet, around 12% of the population in the East Europe is at risk of inadequate zinc intake. The zinc intake of people in South and South-East Asia, Sub-Saharan and North Africa, and East Mediterranean is around 9 mg Zn/day, but only 10–12% of zinc is absorbed because of a low-bioavailability diet (world average ~15%). Therefore, the food supply in these areas provided only 47–60% of zinc requirements (world average 72%) and about 70% people are at risk of insufficient zinc levels in the body. The worst situation is in South Asia, where over 95% of people are at risk of low zinc intake [11]. Infants, children, adolescents, pregnant and lactating women and the elderly are in the highest risk of zinc depletion, as all these groups have increased requirements [12,14].

Zinc plays a very important role in maintaining homeostasis. It is part of around 3000 human proteins, in which it serves as a catalytic, structural, or regulatory ion [3,15,16]. Thus, it plays a crucial role in the proper functioning of cells (including their differentiation, growth and division), endocrine and immune system, transcription, synthesis of proteins, RNA and DNA; and DNA replication [1,8,14]. Zinc is also critical in maintaining the redox balance. It demonstrates antioxidant action and protective effects against reactive oxygen species (ROS) which are synergistic with other antioxidants (e.g., vitamin E). The level of zinc influences the activity of many antioxidant enzymes, including Cu/Zn superoxide dismutase (SOD1), which protects, among others, from DNA damages [1]. Zinc also participates in the metabolism of various microelements [2]. There are studies linking zinc deficiency with the deteriorating vision that comes with ageing [14]. In hippocampus, zinc influences cognitive functions, improves memory and minimizes the risk of depression. In addition, it reduces fatigue, mood swings and psychomotor hyperactivity. It is well known that zinc is crucial in aiding in the production of immune system cells [1,5,14] and in increasing cell sensitivity to insulin [1,17,18]. Zinc is also needed for growth of skin, hair and nails, as it is involved in the proper formation of connective tissues and collagen synthesis [1,9,14]. Therefore, zinc deficiency may lead to severe changes in the functioning of the body, including the reproductive system [1,14].

### 1.2. Role of Zinc in Female Reproductive System 

Zinc is pivotal for the proper functioning of the reproductive system, because the cells of this system differentiate and proliferate extensively, and these processes are zinc-dependent. It plays a critical role in the reproductive system of both sexes, because it is required for spermatozoa development, ovulation, fertilization, normal pregnancy, fetal development, and parturition [6,16,19]. When present at correct levels, it maintains normal homeostasis of testosterone, and male fertility parameters such as sperm count, density, motility, morphology and viability, seminal pH, or semen volume [7,14]. Zinc deficiency in males results in impotence, hypogonadism or delayed sexual development [14]. Moreover, zinc supplementation results in a reduction of prostate size in benign prostate hyperplasia and symptoms of this condition [20,21,22]. 

**Figure 1:**
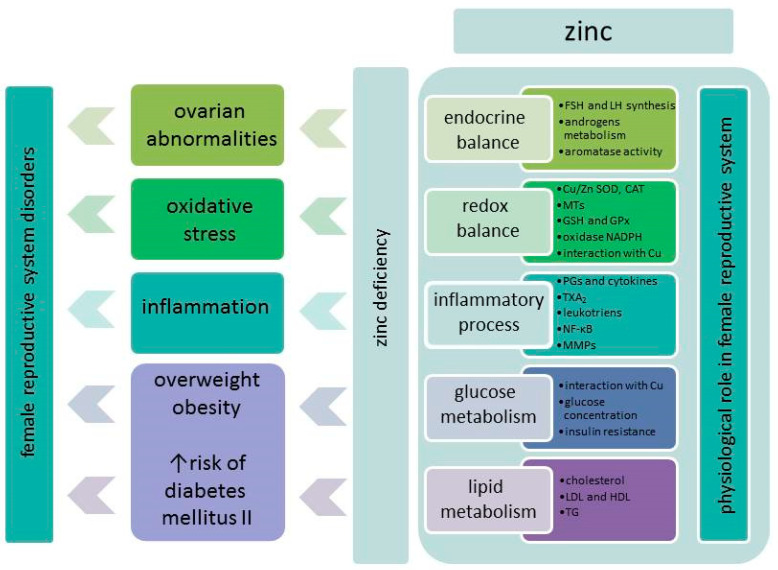
Involvement of zinc in female reproductive system according to [19,25,26,27,28,29,30,31]. Abbreviations: CAT: catalase; Cu/Zn SOD: Cu/Zn superoxide dismutase; FSH: follicle stimulating hormone; GPx: glutathione peroxidase; GSH: glutathione; HDL: high density lipoprotein; LDL: low density lipoprotein; LH: luteinizing hormone; MMPs: matrix metalloproteinases; MTs: metallothioneines; NF-κB: nuclear factor κB; PGs: prostaglandins; TG: triglycerides; TXA_2_: thromboxane A_2_.

In contrast to the male reproductive system, less in known about the effects of zinc on the female reproductive system, as only relatively few investigations have been performed [6,19]. The majority of studies have been focused on the role of zinc and its supplementation on the course of pregnancy and fetal development, which have been extensively reviewed in recent years ([23,24]; among others). Therefore, this issue is beyond the scope of this review. The summary of the basic influence of zinc on female reproductive system is presented on Figure 1.

A number of studies, mainly based on animal research, suggest that zinc deficiency in women could result in a number of pathological conditions: impaired synthesis and/or secretion of follicle-stimulating hormone (FSH) and luteinizing hormone (LH), abnormal ovarian development, disruption of the menstrual cycle, prolonged gestation period, abortion, still-births, gross congenital malformation of fetuses, teratogenic effects, delayed and prolonged deliveries with excessive bleeding, difficult parturition, uncoordinated uterine impulses or inefficient uterine contractions, pre-eclampsia and low birth weights of infants [19,25,26]. Only several human case studies provide data concerning zinc deficiency in women. Ronaghy and Halsted (1975) describe two young Iranian women (aged 19 and 20), who suffered from nutritional dwarfism and sexual infantilism. Their breasts were minimally developed for their age, they had neither axillary nor pubic hair. Moreover, very low plasma and erythrocyte zinc levels were noted. After supplementation with zinc sulfate they menstruated for the first time and developed considerably more breast tissue, as well as the growth of pubic and axillary hair [32]. Same symptoms were reported in several women from Turkish villages [33].

There is no clear association between serum zinc concentrations and infertility. In women demonstrating normal sexual development but long-term infertility with celiac disease low concentrations of zinc in serum have been reported [33], but another study of 48 infertile women found levels of zinc physiological [34]. Ng et al. (1987) did not observe any relationship between zinc concentration in follicular fluid and follicular volume, presence/absence of oocytes in the follicle or determining which oocyte could be fertilized among 33 women undergoing in vitro fertilization [35]. Menezo et al. (2011) report significantly higher zinc concentrations in serum, i.e., nearly twice as high, compared to the follicular fluid of 24 women [36].

Recently, many advances have been made in explicating the crucial role of zinc in oocytes (studies carried out on mouse oocytes), where zinc acts as a regulator of meiosis throughout the entirety of oocyte maturation, including the maintenance of and release from the first and second meiotic arrest points. The first arrest, at prophase I, is maintained by zinc affecting the MOS-MAPK (MOS-mitogen activated protein kinase) pathway [15,37]. During maturation, the total zinc concentration of the oocyte increases. This is needed for the first meiotic division and following metaphase II arrest. In order to activate an oocyte and resume the meiotic cell cycle, a fertilized oocyte rapidly ejects intracellular zinc into the environment, which is called “the zinc spark”. Moreover, zinc homeostasis in the oocyte is regulated by the cumulus cells, which control the timing of the increase in free zinc concentration in the oocyte during maturation [15,37,38,39,40].

Zinc plays a critical role in fertility as it also acts as a cofactor in enzymes of the folate cycle, which are involved in homocysteine recycling to methionine. Human oocytes have limited capacity for recycling, because the cystathionine beta synthase pathway in oocytes is absent and the zinc-dependent betaine homocysteine methyltransferase pathway is poorly expressed [7,36,41]. Homocysteine, which is considered a negative indicator of oocyte quality, is increased during ovarian stimulation, and when it enters the oocyte it can induce defective methylation, oxidative stress, apoptosis and cellular dysfunction by counteracting the action of S-adenosyl methionine [36,41].

## 2. Materials and Methods

This review is based on original papers (mainly randomized, controlled studies on populations of women) published up to the end of May 2020 found in PubMed, Scopus, Google Scholar and Cochrane Library databases. In the search process the following key terms were used: “polycystic ovary syndrome”, “dysmenorrhea” and “endometriosis” with a combination of “trace elements” OR “microelement” OR “Zn” OR “zinc” OR “zinc supplementation” OR “Zn supplementation”. Only the papers written in English were included. The excluding criteria were as follows: secondary dysmenorrhea, co-occurrence of other diseases, in vitro studies, animal research, retracted articles, incomplete/insufficient data. Two authors independently searched the literature and after elimination of duplicate articles evaluated the eligibility of papers according to abovementioned criteria. The other two authors extracted data from each article included in the review.

## 3. Results and Discussion

In this review, 36 studies were included: 17 articles concerned polycystic ovary syndrome (summarized in Table 2 and Table 3), six articles referred to primary dysmenorrhea including one paper describing five case reports (Table 4), eight were about endometriosis and five about pre- and post-menopause.

### 3.1. Zinc and Polycystic Ovary Syndrome (PCOS)

Polycystic ovary syndrome (PCOS) is considered to be the most common endocrine and metabolic disorder in women of reproductive age. PCOS is a heterogeneous disorder connected with symptoms of hormonal imbalances (especially increased androgen concentration) and ovarian dysfunction. Depending on the criteria, the prevalence of PCOS is estimated to be as high as 20% of premenopausal women [42] and even up to 30% among obese women [43]. The pathogenesis of this disease still remains unclear, but PCOS is manifested by: dysregulation in menstrual cycle, hyperandrogenism, insulin resistance and impaired hormonal and lipid balance [44]. 

Table 2 and Table 3 provide an overview of scientific publications describing women with PCOS (diagnosis was based on Rotterdam criteria), in whom serum zinc concentration was measured, and statistically significant changes in various biochemical parameters compared to appropriate controls: healthy women in Table 2, and the PCOS group without supplementation in Table 3. Half of the analyzed studies from Table 2 showed a reduced serum zinc concentration in women with PCOS and most of these changes were statistically significant. Only Kurduglu et al. (2012) noted a significantly higher serum zinc concentration [45]. As shown in Table 2, the most frequently observed changes in women with PCOS were disorders in hormonal, lipid and redox balance, as well as insulin resistance. Zinc, due to its multidirectional effect, may contribute to the development of many of these abnormalities.

One of the main biochemical signs of PCOS is insulin resistance, with compensatory hyperinsulinemia affecting about 70% of patients with PCOS [57]. It is associated with the fact that these women are at increased risk of developing type 2 diabetes mellitus, cardiovascular disease, hypertension and gestational diabetes [58,59,60]. Zinc is important for insulin synthesis, release, action and storage in both, normal and diabetes mellitus conditions [61], because of its regulatory action in phosphorylation signaling of insulin [62] and its effect on oxidative stress. Among the presented studies in Table 2 different biomarkers were used to establish glucose tolerance and insulin resistance. Studies documented increases in HOMA-IR, HOMA2-IR (homeostasis model assessment—insulin resistance index), and also in concentration of insulin, fasting insulin, glucose and fasting glucose [18,27,46,48,49], but decreases in GIR (glucose/insulin ratio) and QUICKI (quantitative insulin sensitivity check index) [18,27]. It was noted that zinc supplementation (Table 3) seems to be beneficial for patients with PCOS, because it was observed to significantly decrease both insulin concentration and HOMA-IR index [52,53]. Hyperinsulinemia and insulin resistance have been linked in the pathogenesis of PCOS and also may contribute to type 2 diabetes mellitus and micro- and macrovascular complications as long-term risks [63,64,65]. Bizoń et al. (2020) conducted a study comparing PCOS women according to BMI value (<25 vs. ≥25), but they did not include control group in their study. Although serum zinc concentration was unchanged, increased serum copper concentration and Cu/Zn ratio in overweight/obese women was noted. In these women also glucose metabolism parameters (except SIRT1—sirtuin 1) were significantly higher. Above disturbances could result from overweight/obesity and insulin resistance [29].

Closely related to insulin resistance is dyslipidemia, characterized in PCOS by elevated triglyceride-rich lipoproteins, bioaccumulation of LDL-cholesterol and decrease of HDL-cholesterol [27,66]. Eight weeks of zinc sulfate supplementation significantly decreased triglyceride, total cholesterol, LDL-cholesterol and VLDL-cholesterol concentrations [52,53], as shown in Table 3.

Women with PCOS often demonstrate hormonal disorders such as hyperandrogenism as well. Such abnormalities were noted by the authors of many studies as significant increases in total testosterone, free testosterone, and dehydroepiandrosterone concentration compared with women without PCOS [27,28,45,46,48] (Table 2), while the effect of zinc supplementation on hormonal balance in women with PCOS, especially on testosterone concentrations, is still unclear (Table 3). Increased peripheral synthesis of androgens, alongside that in adrenals and ovaries are thought to significantly contribute to hyperandrogenism in PCOS [67]. Moreover, PCOS is associated with a distinct increase in global activity of 5α-reductase and this leads to an enhanced conversion of testosterone to dihydrotestosterone in peripheral target cells, which results in androgen actions [67,68]. Zinc is considered an anti-androgen by inhibiting 5α-reductase and thus decreasing the production of dihydrotestosterone [28,69]. Its other involvement in metabolism of androgens includes inhibiting aromatase (and thus decreasing testosterone transformation into estradiol) and increasing conversion of androstenedione to testosterone [28,30,69,70]. In addition, it was shown that zinc deficiency disturbed the activity of angiotensin converting enzyme, which seems to be involved in the synthesis of adrenals androgens [71]. Because DNA-binding domain of androgen receptor is a zinc finger protein, zinc can also affect the action of androgens and zinc deficiency was shown to suppress activity of this receptor [30,71]. One of the effects of hyperandrogenism is hirsutism, which was also observed in women with PCOS [28]. However, no association has been found between hirsutism and serum zinc concentration. Changes in LH, FSH and prolactin concentrations were also associated with hormonal imbalances among women with PCOS, although the direction of these changes was not so obvious [27,46,47,48,50] and in some studies statistically insignificant [28,46,47,48,49,50], also following supplementation with zinc sulfate [54]. 

Zinc is considered as one of the cofactors of antioxidant enzymes, such as catalase (CAT) and SOD1 [72]. While the studies described in this review did not show any changes in SOD1 activity, changes in serum zinc concentration, serum copper concentration, as well as Cu/Zn ratio were observed in woman with PCOS [29,47,51]. Higher generation of ROS is postulated as one of the factors involved in the etiopathogenesis of PCOS [73], because it results in lipid peroxidation and damage of cell membrane lipids [48]. The redox imbalance in PCOS is manifested by an impaired antioxidative mechanism, i.e., decrease of CAT and glutathione peroxidase (GPx) activity, and increase of oxidative stress biomarkers, i.e., malondialdehyde (MDA) concentration [48]. In women with PCOS, decreased lipid peroxidation, indicated by lower MDA concentrations, was observed following zinc supplementation [54,56] (Table 3).

The studies summarized in Table 3 show the positive effect of zinc supplementation on women with PCOS compared with that without supplementation; this was confirmed by an increase in the total antioxidant capacity (TAC) with a simultaneous decrease in protein carboxyl (PCO) and MDA concentrations. In addition, various insulin resistance indices were documented (i.e., diminished insulin concentration), as well as lowered concentrations of lipids, such as total cholesterol and triglycerides. Additionally, a decrease in the concentration of the hormones testosterone and dehydroepiandrosterone (DHEAS) was shown. However, it cannot be affirmed that only zinc may be responsible for these changes, because in some studies, a simultaneous supplementation of zinc with magnesium [55,56], or calcium and vitamin D [56] were also used.

### 3.2. Zinc and Dysmenorrhea 

Dysmenorrhea is a substantial problem in gynecology. About 20–90% of girls and young women aged 10–20 and 8.8% of women aged 19–41 suffer from menstrual pains [74]. Dysmenorrhea is classified as primary and secondary. Primary dysmenorrhea (PD) is defined as a painful menstruation resulting from uterine spasm in the absence of pelvic pathology; it is characterized by recurrent and crampy lower abdominal pain during menstruation. It could also be accompanied by nausea, vomiting and loss of appetite (89%), fatigue (85%), diarrhea (60%), headache (60%), restlessness, insomnia and, rarely, fainting [75,76,77,78]. Secondary dysmenorrhea refers to the same clinical features of pain during menstruation, but can be attributed to pelvic pathology. In this review we focused only on PD.

The etiopathogenesis of PD has been primarily associated with the activity of prostaglandins and leukotrienes. Prostaglandins (e.g., PGF2-α) temporarily limit or stop the blood supply to uterus by stimulating its contraction, which reduces the amount of blood perfusing the uterus through myometrial compression of the blood vessels [71]. This deprives the uterus of oxygen, which results in cramping and abdominal pain. Higher concentrations of PGF2-α and leukotrienes in menstrual blood and in uterine smears were observed in women with signs of painful menstruation [75,79,80]. Zinc reduces the synthesis of prostaglandins through its ability as an endogenous antioxidant catalyst and an anti-inflammatory agent that can improve microcirculation of endometrium tissue [81,82]. In vivo studies indicate that zinc supplementation decreases the activity of cyclooxygenase-2 (COX-2) [82,83]. Moreover, patients with signs of premenstrual syndrome demonstrated lower concentrations of zinc in the luteal phase than in other phases of menstrual cycle compared with controls, which can indicate zinc deficiency [84]. Zinc could also prevent spasms and pain by its antioxidant and anti-inflammatory actions, by influencing SOD1 [85]. This metal takes part in the regulation of chronic inflammatory status through the reduction of inflammatory cytokines [86]. Therefore, it could be concluded that zinc supplementation may be a protective factor for uterine muscle cells (Table 4).

The effects of zinc supplementation in women with PD, which are based predominantly on randomized studies, are presented in Table 4. Only women suffering from PD having regular menstrual cycles (21–35 days) participated in these studies, excluding ones with gynecological disease or disorders (especially secondary dysmenorrhea) and significant medical history. In addition, all patients were subjected to pelvic ultrasonography by gynecologist. Only work by Eby (2007) described five case reports concerning PD [87]. Based on the data in Table 4 it is possible that zinc supplementation may be used in preventing PD. Women were supplemented with zinc at doses of 20 to 126 mg/day, for 3–6 days, usually for two or three menstruation cycles. Intensity of menstrual pain was assessed on a scale of 1–10 using two methods: Visual Analog Scale (VAS) and Pain Visual Analog Scale (PVAS). Pain alleviation was observed in the first cycle of zinc supplementation, but the most significant effect was observed in the second or third ones [88,89,90,92]. Reduction of pain severity was noted independently of zinc dose (in all used doses and without any adverse effects) administered for 3–6 days starting before or during menstrual cycle. It seems that zinc dose above 30 mg/day does not improve the efficacy of supplementation [88,91]. This is particularly important, because the daily dose of zinc in dietary supplements, according to EFSA and IOM recommendations, should not exceed 25 and 40 mg, respectively [9,10]. Interestingly, greater pain alleviation was observed after simultaneous administration of zinc with mefenamic acid than with mefenamic acid alone [91].

However, in contrast to the dose, significant alleviation of menstrual pain likely depends on the regularity of zinc supplementation. The best results were obtained when zinc was administered for each menstrual cycle for 3–6 days before and during menstruation [87,89,92]. However, in one study alleviation of menstrual pain was obtained, when zinc was administered for four days before menstruation [90]. Thus, this effect seems also to be dependent on time of administration. Unfortunately, plasma zinc concentration was not assessed as a biomarker of supplementation in the described studies, which prevents accurate determination of the effective zinc dose. In addition, the potential interaction between zinc and copper was not taken into account.

### 3.3. Zinc and Endometriosis

Endometriosis is a debilitating gynecologic disease characterized by the implantation of endometrial tissue in ectopic locations, including the pelvic peritoneum, ovaries, and bowel. The prevalence of endometriosis in reproductive aged women is in the range of 2–10% [93] and as high as 35–50% in women with pain and/or unexplained infertility [94]. This disease is a major cause of disability and significantly compromised quality of life in adolescents and adult women. Its symptoms include dysmenorrhea, dyspareunia, lower abdominal and/or back pain, dyschezia, dysuria, and altered bowel habits [95]. Endometriosis is a major cause of infertility due to the inflammation-associated reductions in oocyte quality and endometrial receptivity to embryonic implantation [96]. Despite the high prevalence of the disease, not much is known about its etiology, possible risk factors, and an adequate and satisfactory therapy.

Among many candidate factors implicated in the pathophysiology of endometriosis, oxidative stress, prostaglandins, cytokines and matrix metalloproteinases (MMPs) have been proposed to play a key role [97]. Thus, it is suggested that antioxidants, including zinc, may play a role in endometriosis due to its function as an antioxidant, anti-inflammatory and immune regulation factor. Some clinical studies report lowered serum zinc concentrations in women with endometriosis, suggesting that zinc maybe involved in the multifactorial pathogenesis of this disease [98,99,100,101]. Furthermore, it has been noted that women with endometriosis might experience increased oxidative stress parameters [102]. A significant reduction of SOD1 and increase of lipid peroxidases in plasma of women with endometriosis has been reported [103,104]. 

It is possible that zinc may play an important role in endometriosis because it is also an inhibitor of MMPs. The elevated concentrations of MMP-2 [105] and MMP-9 [98] have been reported in women with endometriosis versus controls. Moreover, advanced endometriosis is correlated with higher MMP-2 expression [106]. The levels of MMP-3 mRNA were significantly higher in cases of advanced stage (II–IV) of endometriosis than in control group [107]. Although the etiology of endometriosis needs to be clarified and many factors (e.g., smoking, alcohol consumption) are involved in its development, research in last years focused on the possible role of ROS [99,108,109,110]. In view of the above, there are only few studies supporting clinical effectiveness of supplementation of zinc and other antioxidants in endometriosis [108,111], but to provide strong evidence more randomized, placebo-controlled trials are needed. 

### 3.4. Zinc in Pre- and Post-Menopause

The effects of zinc on the reproductive system later in life (pre- and post-menopausal periods) is virtually unknown. However, few studies indicate that zinc may play a role in maintaining the composition of the vaginal extracellular matrix [112,113]. Zinc is known to play a part in collagen metabolism by diminishing the activity of lysyl oxidase, which participates in the formation of cross bonds in the process of collagen synthesis [114]. 

Takcas et al. (2020) did not identify any relationship between daily oral zinc supplementation (30 mg) and zinc concentration in cervicovaginal lavage fluid in pre- and post-menopausal women [113]. Another pilot study found zinc contained in vaginal moisturizer gel to induce significant improvement of postmenopausal vulvovaginal symptoms [112]; however, these findings need to be confirmed in larger studies.

In another study, an attempt was made to establish the influence of estrogen and estro-progestin therapy in pre- and post-menopausal women on the concentration of essential metals, including zinc. Results indicate that hormonal therapy did not influence blood and serum zinc concentrations in postmenopausal women, although these levels were higher than in the premenopausal group [115]. Additionally, Sunar et al. (2008) did not demonstrate any significant effect on serum estradiol and progesterone concentration among postmenopausal women after two weeks supplementation with low doses of zinc [116].

## 4. Conclusions

**Figure 2:**
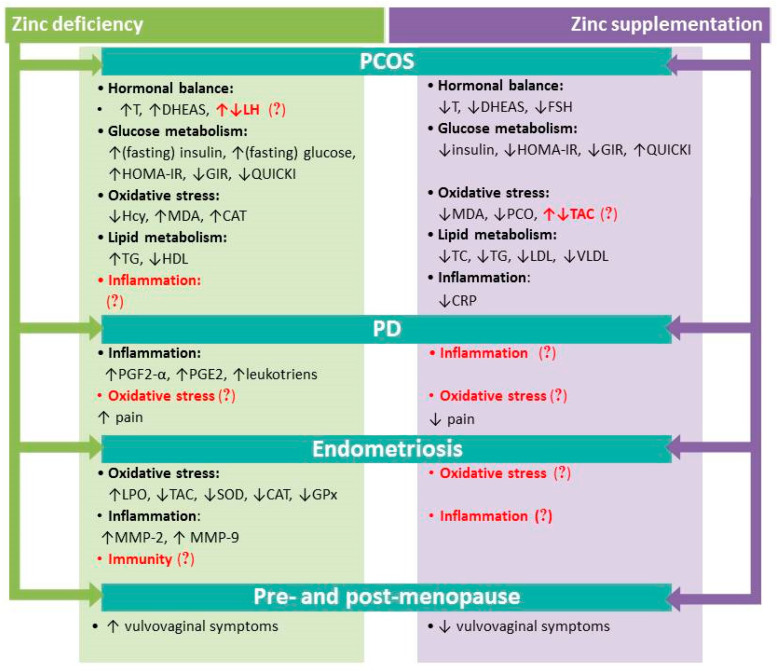
Summary of the effects of zinc deficiency and zinc supplementation in PCOS, PD, endometriosis and menopause. Question marks indicate uncertainty/gaps requiring elucidation and further research. Abbreviations: CAT: catalase; CRP: C-reactive protein; DHEAS: dehydroepiandrosterone sulfate; FSH: follicle stimulating hormone; GIR: glucose/insulin ratio; GPx: glutathione peroxidase; Hcy: homocysteine; HDL: high density lipoprotein; HOMA-IR: homeostasis model assessment – insulin resistance index; LDL: low density lipoprotein; LH: luteinizing hormone; MDA: malondialdehyde; MMP: matrix metalloproteinase; PGF2-α: prostaglandin 2α; PGE2: prostaglandin E2; PCO: protein carbonyl; QUICKI: quantitative insulin sensitivity check index; SOD: superoxide dismutase; T: testosterone; TAC: total antioxidant capacity; TC: total cholesterol in serum; TG: triglycerides; VLDL: very low density lipoprotein.

In summary, zinc is important in both the male and female reproductive system. It plays a critical role in the functioning of this system by serving a protective function, e.g., as an antioxidant. Zinc supplementation seems to improve PCOS symptoms, particularly among women with dysregulated insulin resistance and lipid balance. In addition, reduced levels of zinc in PCOS are accompanied by impaired hormonal, lipid and glucose metabolism and increased concentrations of oxidative stress biomarkers. In PD, zinc administered in one to four daily doses of 20–30 mg before and during each menstrual cycle may reduce the intensity of pain accompanying menstruation. On the other hand, possible involvement of zinc in the pathogenesis of endometriosis needs to be clarified, as does its role in alleviating this disorder (Figure 2). 

It is still too early to draw more explicit conclusions and further studies explaining the potential impact of zinc and its supplementation on female reproductive system would be highly advisable and valuable. The role of zinc in endometriosis and in postmenopausal women is an emerging issue that also requires more attention and scientific research.

## Figures and Tables

**Table 1:** Zinc dietary recommendation according to WHO/FAO, IOM and EFSA [8,9,10].

WHO/FAO				IOM		EFSA	
Age, Sex	RNI (mg/Day)			Age, Sex	RDA (mg/Day)	Age, Sex	PRI (mg/Day)
	High^a^	Moderate^b^	Low^c^				
0–6 months	1.1^d^	2.8^e^	6.6^f^	0–6 months	2 (AI)		
7–12 months	0.8^d^; 2.5^g^	4.1	8.4	7–12 months	3	7–11 months	2.9
1–3 years	2.4	4.1	8.3	1–3 years	3	1–3 years	4.3
4–6 years	2.9	4.8	9.6	4–8 years	5	4–6 years	5.5
7–9 years	3.3	5.6	11.2	9–13 years	8	7–10 years	7.4
						11–14 years	9.4
10–18 years				14–18 years		15–17 years	
Males	5.1	8.6	17.1	Males	11	Males	12.5
Females	4.3	7.2	14.4	Females	9	Females	10.4
≥19 years				≥19 years		≥18 years	
Males	4.2	7.0	14.0	Males	11	Males	
						300^h^	9.4
						600^h^	11.7
						900^h^	14.0
						1200^h^	16.3
Females	3.0	4.9	9.8	Females	8	Females	
						300^h^	7.5
						600^h^	9.3
						900^h^	11.0
						1200^h^	12.7
Pregnancy				Pregnancy		Pregnancy	+1.6
1st trimester	3.4	5.5	11.0				
2nd trimester	4.2	7.0	14.0	14–18 years	12		
3rd trimester	6.0	10.0	20.0	19–50 years	11		
Lactation				Lactation		Lactation	+2.6
0–3 months	5.8	9.5	19.0				
3–6 months	5.3	8.8	17.5	14–18 years	13		
6–12 months	4.3	7.2	14.4	19–50 years	12		

^a^ high bioavailability of dietary zinc (50%); ^b^ moderate bioavailability of dietary zinc (30%); ^c^ low bioavailability of dietary zinc (15%); ^d^ exclusively human-milk-fed (bioavailability of zinc—80%); ^e^ infants fed whey-adjusted milk formula and to partly human-milk-fed or given low-phytate feeds supplemented with other liquid milks; ^f^ infants fed a phytate-rich vegetable protein-based formula with or without whole-grain cereals; ^g^ not applicable to infants consuming human milk only; ^h^ level of phytate intake (mg/day); AI—adequate intake; PRI—population reference intake; RDA—recommended dietary allowance; RNI—recommended nutrient intake.

**Table 2:** Characteristics of women with polycystic ovary syndrome (PCOS), their serum zinc concentration and the statistically significant changes in various biochemical parameters.

Age (Years)		BMI (kg/m^2^)		Zinc (µg/dL)		Additional Information			Ref.
Control	PCOS	Control	PCOS	Control	PCOS	Parameters	Control	PCOS	
27.75 ± 5.45 (*n* = 30)	24.25 ± 4.68 (*n* = 35)	22.63 ± 3.08	21.72 ± 3.02	77 ± 19	92 ± 20^b^	T (ng/dL) DHEAS (µg/dL)	45.8 ± 13.4 180.85 ± 80.45	83.24 ± 37.04^c^ 307.81 ± 132.77^c^	[45]
28.33 ± 0.87 (*n* = 46)	28.93 ± 0.36 (*n* = 132)	23.02 ± 0.17	23.57 ± 0.49	56 ± 6.7	60 ± 34.4	LH (mIU/mL)	7.39 ± 0.41	5.91 ± 0.39^a^	[46]
						T (ng/dL)	47 ± 24	149 ± 18^c^	
						fasting insulin (µIU/mL)	8.12 ± 0.66	12.38 ± 0.83^b^	
						HOMA2-IR	1.27 ± 0.37	2.21 ± 0.25^c^	
						manganese (µg/dL)	13 ± 1.1	23 ± 2.2^b^	
						calcium (µg/dL)	210 ± 11	300 ± 42^a^	
28.0 ± 5.9 (*n* = 33)	25.4 ± 6.7 (*n* = 53)	23.5 ± 4.9	27.4 ± 6.8^a^	78.1 ± 14.7	66.3 ± 13.2^c^	HDL-C (mg/dL)	52.4 ± 11.3	45.6 ± 8.9^b^	[47]
						TG (mg/dL)	79.3 ± 39.3	109.9 ± 60^a^	
						TG/HDL ratio	1.6 ± 1	2.5 ± 1.7^a^	
						Hcy (µmol/L)	10.5 ± 2.8	8.9 ± 2.3^a^	
*29* *(26–31)* (*n* = 105)	*27.0* *(25–30)* (*n* = 96)	*20.45* *(18.98–22.50)*	*22.07* *(19.72–25.03)* ^c^	*87.74* *(79.21–96.63)*	82.37 (76.96–89.45)^b^	LH (mIU/mL)	*4.38 (3.14–5.61)*	*9.80 (5.36–15.14)^c^*	[27]
						LH/FSH	*0.64 (0.46–0.81)*	*1.51 (0.90–2.17)^c^*	
						tT (ng/dL)	*33.98 (25.92–44.93)*	*55.01 (44.06–69.98)^c^*	
						DHEAS (µg/dL)	*270.94 (228.21–320.37)*	*311.03 (269.96–348.59)^c^*	
						SHGB (nmol/L)	*37.20 (28.90–57.81)*	*16.74 (9.55–48.03)^c^*	
						fasting insulin (µIU/mL)	*6.90 (5–8.60)*	*10.35 (6.43–13.50)^c^*	
						fasting glucose (mg/dL)	*83.52 (77.94–88.92)*	*86.49 (80.82–92.12)^a^*	
						GIR	*12.04 (9.42–15.65)*	*8.61 (6.62–13.33)^c^*	
						HOMA-IR	*1.44 (1–1.84)*	*2.22 (1.35–2.97)^c^*	
						QUICKI	*0.36 (0.35–0.38)*	*0.34 (0.32–0.37)^c^*	
						TG (mg/dL)	*7.54 (5–9.91)*	*9.91 (7.1–14.74)^c^*	
						HDL-C (mg/dL)	*56.5 (47.7–66.9)*	*53.5 (45.4–60.8)^a^*	
29.4 ± 8.8 (*n* = 53)	26.2 ± 5.5 (*n* = 71)	23.8 ± 4.7	26.9 ± 5.4	99.4 ± 19.9	84.4 ± 25.5^a^	glucose (mg/dL)	78.5 ± 7.1	90.2 ± 6.1^b^	[48]
						insulin (µIU/mL)	9.7 ± 6.4	13.18 ± 8.9^a^	
						HOMA-IR	1.88 ± 1.68	2.92 ± 2.15^a^	
						LH (mIU/mL)	4.48 ± 2.03	7.95 ± 5.06^c^	
						tT (ng/dL)	89 ± 34	155 ± 91^c^	
						freeT (ng/dL)	146 ± 22	231 ± 54^c^	
						MDA (nmol/mL)	3.1 ± 2.7	23.0 ± 13.8^c^	
						catalase (µg/mL)	2302 ± 437.3	1875.7 ± 435.1^a^	
						GPx (µg/mL)	3.2 ± 0.5	3.6 ± 0.4^a^	
*33(30–37)* (*n* = 559)	*30.0(28–33)* (*n* = 578)^c^	*21.12* *(19.72–23.34)*	*22.01* *(19.97–24.44)^c^*	*646.23* *(541.25–754.97)*	*659.42* *(560.82–771.16)*	FSH (mIU/mL)	*7.90 (6.67–9.18)*	*6.90 (6.01–8.16)^c^*	[47]
						LH (mIU/mL)	*3.68 (2.82–4.86)*	*5.59 (3.91–9.84)^c^*	
						copper (µg/dL)	*114.22 (98.34–138.79)*	*126.19 (106.91–152.27)^c^*	
						calcium (mg/dL)	*6.44 (5.76–7.04)*	*6.2 (5.64–6.84)^c^*	
25.34 ± 5.82 (*n* = 67)	24.06 ± 6.12 (*n* = 65)	25.27 ± 2.68	26.0 ± 4.52	89.22 ± 9.83	95.45 ± 10.94	hirsutism score	*1.28* ± 1.73	*8.32* ± 3.67^a^	[49]
						insulin (µIU/mL)	6.48 ± 2.34	10.71 ± 5.47^a^	
						HOMA-IR	1.92 ± 0.58	2.37 ± 0.77^a^	
25.18 ± 3.1 (*n* = 40)	25.48 ± 3.56 (*n* = 40)	28.28 ± 8.33	28.34 ± 7.17	83.8 ± 10.11	75.31 ± 33.91	prolactin (IU/mL)	9.19 ± 2.2	15.07 ± 8.42^c^	[50]
						LH (IU/mL)	4.57 ± 1.8	6.48 ± 4.36^a^	
27.1 ± 4.8 (*n* = 50)	26.9 ± 5.2 (*n* = 50)	25.6 ± 5.7	28.4 ± 4.2^b^	*138* *(98.2–192)*	*123* *(103.5–180.7)*	Cu/Zn	*1.5 (0.9–1.8)*	*1.1 (0.8–1.4)^a^*	[51]
29.17 ± 5.03 (*n* = 90)	28.68 ± 5.08 (*n* = 60)	27.92 ± 4.70	29.14 ± 5.57	108.31 ± 63.29	81.33 ± 24.28^a^	insulin (µIU/mL)	11.80 ± 5.12	15.67 ± 7.88^b^	[18]
						HOMA-IR	2.46 ± 1.15	3.44 ± 2.11^b^	
						QUICKI	0.339 ± 0.02	0.326 ± 0.03^b^	
						GIR	8.63 ± 4.36	7.05 ± 4.77^b^	
15.21 ± 1.42 (*n* = 51)	15.67 ± 1.58 (*n* = 34)	23.0 ± 3.23	25.79 ± 4.80	101.72 ± 16.71	102.27 ± 10.41		“idiopathic hirsutism" group		[28]
						T (ng/dL)	30.57 ± 10.61	54.71 ± 26.54^a^	
						DHEAS (µg/dL)	273.43 ± 61.07	283.21 ± 142.87^c^	
BMI < 25									[29]
	*24* *(18–39)* (*n* = 55)		*20.94* *(16.16–24.89)*		*85.77* *(58.97–107.57)*	copper (µg/dL)		*80.21 (38.63–139.50)*	
						Cu/Zn		*0.96 (0.62–1.50)*	
						glucose (mg/dl)		*87 (75–98)*	
						glucose 120′ (mg/dL)		*92 (31–141)*	
						insulin (µIU/mL)		*4.71 (1.72–13.11)*	
						insulin 120′(µIU/mL)		*24.47 (8.36–90.46)*	
						HOMA-IR		*1.05 (0.36–2.88)*	
						SIRT1 (ng/mL)		*1.18 (0.81–30.87)*	
BMI ≥ 25									
	*23* *(17–38)* (*n* = 21)		*31.14* *(25.10–41.52)*		*81.82* *(59.60–109.99)*	copper (µg/dL)		*94.82 (61.99–118.87)*	
						Cu/Zn		*1.06 (0.73–1.67)*	
						glucose (mg/dl)		*88 (79–116)*	
						glucose 120′ (mg/dL)		*113 (75–185)*	
						insulin (µIU/mL)		*10.32 (2.73–34.67)*	
						insulin 120′ (µIU/mL)		*52.2 (18.21–151.14)*	
						HOMA-IR		*2.17 (0.61–9.93)*	
						SIRT1 (ng/mL)		*1.09 (0.88–3.59)*	

^a^*p* ≤ 0.05 vs. control; ^b^ *p* ≤ 0.01 vs. control; ^c^ *p* ≤ 0.001 vs. control; Values are expressed as mean ± SD; mean ± SEM (underlined); *medians (25–75% quartiles) (italics)*; *medians (min–max value) (italics, underlined)*; DHEAS: dehydroepiandrosterone sulfate; freeT: free testosterone; FSH: follicle stimulating hormone; GIR: glucose/insulin ratio; **GPx:** glutathione peroxidase; Hcy: homocysteine; HDL-C: high density lipoprotein-cholesterol; HOMA2-IR: homeostasis model assessment—insulin resistance index (HOMA2-IR > 2.1—insulin resistance); HOMA-IR: homeostasis model assessment—insulin resistance index (HOMA-IR > 2.5 was accepted as insulin resistance); LH: luteinizing hormone; QUICKI: quantitative insulin sensitivity check index, SHGB: sex hormone binding globulin; SIRT1: sirtuin 1; T: testosterone; TG: triglycerides; tT: total testosterone.

**Table 3:** The effects of zinc supplementation on various biochemical parameters in women with PCOS.

Type of Supplementation	Inclusion Criteria	Parameters	Placebo			Zinc Supplementation			Ref.
			Baseline	End of Trial	Change	Baseline	End of Trial	Change	
220 mg of zinc sulfate (50 mg Zn) 8 weeks;	PCOS women 20–45 years Placebo group (*n* = 30) Zinc group (*n* = 35)	zinc (µg/dL)	78.25 ± 0.87	79.30 ± 0.85	1.05 ± 0.70	76.11 ± 1.06	108.18 ± 1.77^c^	32.06 ± 1.76^c^	[52]
		insulin (µU/mL)	17.93 ± 1.64	17.97 ± 1.56	0.04 ± 0.23	18.73 ± 1.41	16.19 ± 1.32^c^	−2.54 ± 0.40^c^	
		HOMA-IR	4.16 ± 0.37	4.16 ± 0.36	−0.003 ± 0.05	4.34 ± 0.31	3.71 ± 0.29^c^	−0.62 ± 0.09^c^	
		TC (mg/dL)	196.0 ± 1.5	196.6 ± 1.5	0.66 ± 0.44	196.3 ± 1.7	194.8 ± 1.5^b^	−3.73 ± 1.81^b^	
		LDL-C (mg/dL)	133.2 ± 4.5	134.04 ± 4.4^a^	1.06 ± 0.48	132.77 ± 4.0	130.71 ± 3.7^c^	−5.84 ± 4.41	
		TG (mg/dL)	180.5 ± 9.3	180.4 ± 9.4	−0.33 ± 0.44	182.9 ± 9.2	178.1 ± 8.5^c^	−11.46 ± 3.37^b^	
		T (ng/dL)	90 ± 6	89 ± 6	−1 ± 2	95 ± 6	88 ± 7^a^	−7 ± 3	
		DHEAS (µg/dL)	150 ± 14	147 ± 14	−3 ± 1	156 ± 15	154 ± 14	−19 ± 5^b^	
220 mg of zinc sulfate (50 mg Zn) 8 weeks;	PCOS women 18–40 years Placebo group (*n* = 26) Zinc group (*n* = 26)	zinc (mg/dL)	101.0 ± 14.8	96.9 ± 10.9	−4.1 ± 16.7	113.4 ± 21.3	129.0 ± 30.1^a^	15.6 ± 21.8^c^	[53]
		FPG (mg/dL)	92.5 ± 7.1	93.0 ± 7.5	0.5 ± 6.0	99.8 ± 10.3	95.5 ± 8.0^a^	−4.3 ± 9.6^a^	
		insulin (µIU/mL)	10.0 ± 8.3	11.5 ± 8.8	1.5 ± 8.4	10.4 ± 3.7	7.4 ± 2.0^a^	−3.0 ± 2.9^b^	
		HOMA-IR	2.3 ± 1.9	2.6 ± 2.1	0.3 ± 1.9	2.6 ± 1.2	1.8 ± 0.8^a^	−0.8 ± 0.8^b^	
		HOMA-B	36.4 ± 33.4	41.3 ± 33.4	4.9 ± 32.1	33.7 ± 11.4	23.1 ± 7.4^a^	−10.6 ± 9.5^a^	
		QUICKI	0.35 ± 0.03	0.35 ± 0.04	−0.004 ± 0.05	0.33 ± 0.02	0.35 ± 0.02^a^	0.02 ± 0.02^a^	
		TG (mg/dL)	108.7 ± 50.2	123.2 ± 61.3^a^	14.5 ± 25.3	126.8 ± 56.2	111.2 ± 57.8	−15.6 ± 40.3^b^	
		VLDL-C (mg/dL)	21.7 ± 10.0	24.6 ± 12.3^a^	2.9 ± 5.1	25.4 ± 11.2	22.2 ± 11.6	−3.2 ± 8.1^b^	
220 mg of zinc sulfate (50 mg Zn) 8 weeks	PCOS women 18–40 years Placebo group (*n* = 24) Zinc group (*n* = 24)	zinc (mg/dL)	102.66 ± 13.71	99.32 ± 10.31	−3.34 ± 15.83	111.89 ± 20.9	128.87 ± 30.91	16.98 ± 22.3^c^	[54]
		FSH (IU/L)	7.16 ± 1.82	8.05 ± 1.84	0.89 ± 2.28	12.94 ± 8.52	11.87 ± 7.44	−1.07 ± 5.16^b^	
		freeT (ng/dL)	0.351 ± 0.186	0.341 ± 0.193	−0.010 ± 0.096	0.265 ± 0.115	0.251 ± 0.109	−0.014 ± 0.062^a^	
		17-OHP (ng/mL)	2.10 ± 1.23	1.92 ± 1.35	−0.18 ± 1.78	1.13 ± 0.99	1.0 ± 0.61	−0.13 ± 1.20^c^	
		TAC (nmol/L)	718.2 ± 138.32	666.85 ± 135.7	−51.35 ± 182.81	801.74 ± 210.59	781.66 ± 142.26	−20.08 ± 156.82^b^	
		MDA (nmol/mL)	4.96 ± 2.97	7.30 ± 4.37^a^	2.34 ± 5.53	4.37 ± 1.04	4.28 ± 0.65	−0.09 ± 1.31^c^	
250 mg of magnesium oxide + 220 mg of zinc sulfate (50 mg Zn) 12 weeks; twice a day	PCOS women 18–40 years Placebo group (*n* = 30) Zinc group (*n* = 30)	magnesium (mg/dL)	1.81 ± 0.32	1.76 ± 0.31	−0.05 ± 0.17	1.84 ± 0.29	2.05 ± 0.31	0.21 ± 0.24^c^	[55]
		zinc (mg/dL)	84.9 ± 11.6	84.3 ± 11.1	−0.6 ± 3.9	80.4 ± 12.4	86.3 ± 13.4	6.6 ± 5.0^c^	
		hs-CRP (mg/L)	5.1 ± 1.9	5.2 ± 1.9	0.1 ± 0.7	4.4 ± 2.6	2.8 ± 1.4	−1.6 ± 2.4^c^	
		TAC (nmol/L)	795 ± 132.5	793.5 ± 172.8	−1.5 ± 141.5	712.7 ± 64	785.4 ± 73.1	60.7 ± 69.4^c^	
		PCO (nmol/mg protein)	2.67 ± 0.64	2.70 ± 35	0.02 ± 0.07	2.59 ± 0.39	2.45 ± 0.41	−0.14 ± 0.28^b^	
100 mg of Mg + 4 mg of Zn + 400 mg of Ca + 200 IU of Vit. D; 12 weeks; twice a day	PCOS women 18–40 years Placebo group (*n* = 30) Zinc group (*n* = 30)	magnesium (mg/dL)	1.6 ± 0.3	1.5 ± 0.2	−0.1 ± 0.3	1.5 ± 0.2	1.6 ± 0.2	0.1 ± 0.1^b^	[56]
		zinc (mg/dL)	101.0 ± 14.9	100.1 ± 13.6	−0.9 ± 7.6	106.1 ± 17.9	109.1 ± 18.5	3.0 ± 8.8	
		calcium (mg/dL)	9.3 ± 0.5	9.3 ± 0.8	−0.01 ± 0.6	9.5 ± 0.5	9.9 ± 0.5	0.4 ± 0.3^c^	
		25-OH D (ng/mL)	10.8 ± 4.6	10.9 ± 4.5	0.1 ± 0.5	10.1 ± 4.9	18.0 ± 10.3	7.9 ± 8.4^c^	
		tT (ng/dL)	140 ± 90	150 ± 90	10 ± 40	160 ± 60	140 ± 50	−20 ± 50^a^	
		mF-G scores	12.6 ± 3.9	12.5 ± 3.9	−0.1 ± 0.4	14.3 ± 3.8	11.9 ± 3.1	−2.4 ± 1.2^c^	
		hs-CRP (mg/L)	3.1 ± 2.1	3.3 ± 1.8	0.2 ± 0.8	3.7 ± 1.6	3.0 ± 1.2	−0.7 ± 0.8^c^	
		TAC (nmol/L)	881.6 ± 165.0	873.9 ± 189.3	−7.7 ± 130.1	794.9 ± 59.3	841.5 ± 75.8	46.6 ± 66.5^a^	
		MDA (nmol/mL)	2.2 ± 0.6	2.4 ± 0.9	0.2 ± 1.0	2.8 ± 0.2	2.4 ± 0.2	−0.4 ± 0.3^b^	

^a^*p* ≤ 0.05 vs. control; ^b^ *p* ≤ 0.01 vs. control; ^c^ *p* ≤ 0.001 vs. control; Values are expressed as mean ± SD; mean ± SEM (underlined); 17-OHP: 17-hydroxyprogesterone; DHEAS: dehydroepiandrosterone sulfate; FPG: fasting plasma glucose; freeT: free testosterone; FSH: follicle stimulating hormone; HOMA-B: homeostatic model assessment-beta cell function; HOMA-IR: homeostatic model of assessment-insulin resistance; hs-CRP: high-sensitivity C-reactive protein, LDL-C: low density lipoprotein-cholesterol; MDA: malondialdehyde; mF-G scores: modified Ferriman Gallwey; PCO: protein carbonyl; QUICKI: quantitative insulin sensitivity check index; T: testosterone; TAC: total antioxidant capacity; TC: total cholesterol in serum; TG: triglycerides; tT: total testosterone; VLDL-C: very low density lipoprotein-cholesterol.

**Table 4:** The effects of zinc supplementation on various clinical signs in women with primary dysmenorrhea (PD).

Type of Supplementation	Inclusion Criteria	Parameters	Placebo		Zinc			Ref.
			Baseline	End of Trial	Baseline	End of Trial	*p* Value	
30 mg of zinc (zinc gluconate) 4 days per menstrual cycle	Women with PD 17 years Zinc group (*n* = 1)	Muscle pain			Yes	No		[87]
		Duration of menstrual bleeding			Normal	Normal		
14 mg of zinc (zinc acetate) as throat lozenges 2 days prior to and 1st day of menstrual cycle; 9 × day (total daily dose 126 mg)	Women with PD 23 years Zinc group (*n* = 1)	Muscle pain			Yes	No		
		Menstrual cramp			Yes	No		
		Duration of menstrual bleeding			Normal	Normal		
60 mg of zinc (zinc gluconate) 3–4 days per menstrual cycle; twice a day per 3 cycles	Women with PD 49 years Zinc group (*n* = 1)	Muscle pain			Yes	No		
		Menstrual cramp			Yes	No		
		Duration of menstrual bleeding			Long (8–9 days)	Normal (5 days)		
30 mg of zinc 1–2 days per menstrual cycle;	Women with PD 30 years Zinc group (*n* = 1)	Muscle pain			Yes	No		
		Menstrual cramp			Yes	No		
		Duration of menstrual bleeding			Normal	Normal		
30 mg of zinc 3–5 days per menstrual cycle; 10 years	Women with PD 38 years Zinc group (*n* = 1)	Muscle pain			Yes	No		
		Menstrual cramp			Yes	No		
		Duration of menstrual bleeding			Normal	Normal		
90 mg of zinc (220 mg of zinc sulfate) 4 days—from the day before to the third day of menstrual bleeding per 2 cycles	Women with PD 15–18 years Zinc group (*n* = 56) Control group – placebo (*n* = 46) Randomized Placebo-Controlled Trial	PVAS						[88]
		Severity of dysmenorrhea	7.76 ± 1.30		8.01 ± 1.12			
		1st cycle		7.13 ± 1.30		6.18 ± 1.70	<0.001	
		2nd cycle		6.95 ± 1.67		3.12 ± 1.2	<0.001	
		Diarrhea						
		1st cycle		1 (2.2)*		1 (1.9)		
		2nd cycle		1 (2.4)*		2 (3.8)		
		Headache						
		1st cycle				1 (1.19)*		
		2nd cycle						
		Heartburn						
		1st cycle		1 (2.2)*		1 (1.19)*		
		2nd cycle		1 (2.4)*		1 (1.19)*		
		Duration of menstrual bleeding	6.1 ± 1.02		6.7 ± 1.14			
20 mg of zinc (50 mg of zinc sulfate) 4 days—from the first day to next three days of menstrual bleeding per 3 cycles	Women with PD 14–18 years Zinc group (*n* = 60) Control group—placebo (*n* = 60) DBRCT	VAS						[89]
		Pain duration			5.47 ± 1.32			
		1st cycle		4.62 ± 2.02		3.95 ± 1.52	<0.05	
		2nd cycle		4.43 ± 1.76		3.22 ± 1.35	<0.001	
		3rd cycle		4.42 ± 1.73		2.77 ± 1.47	<0.001	
		Pain severity			7.30 ± 2.43			
		1st cycle		6.96 ± 1.56		6.60 ± 3.79		
		2nd cycle		6.68 ± 1.79		5.01 ± 1.70	<0.001	
		3rd cycle		6.58 ± 1.60		4.23 ± 1.69	<0.001	
50 mg of zinc 4 days—before the menstruation twice a day per cycle	Women with PD 14–18 years Zinc group (*n* = 34) Control group—placebo (*n* = 32) DBRCT	VAS						[90]
		Severity of bleeding	81.2 ± 71.2	78.9 ± 60.5	78.2 ± 54.1	64.3 ± 32.5	<0.05	
		Severity of dysmenorrhea	56.3 ± 15.0	54.5 ± 18.1	64.9 ± 16.2	41.5 ± 22.3	<0.01	
		Muscular pain	23*	22*	27*	20*	<0.03	
		Disability in daily activities	29*	26*	27*	10*	<0.001	
		Weakness	26*	21*	33*	26*	<0.02	
90 mg of zinc (220 mg of zinc sulfate) and 250 mg of mefenamic acid 6 days—three days before and three days after menstruation per 3 cycles	Women with PD 18–26 years Zinc group and mefenamic acid (*n* = 100) Control group—placebo and mefenamic acid (*n* = 100) DBRCT	VAS						[91]
		Mean pain	5.8 ± 2.1	2.9 ± 2.6	5.3 ± 1.8	1.2 ± 1.9	<0.001	
		Dysmenorrhea						
		Yes		67*		36*	<0.001	
30 mg of zinc 2 days before—and continuing until prior to the end of menstrual bleeding twice a day per 3 cycles.	Women with PD 17–25 years Zinc group (*n* = 34)	VAS						[92]
		Menstrual pain			4.92 ± 1.80			
		1st cycle				3.37 ± 2.04	<0.001	
		2nd cycle				3.30 ± 1.93	<0.001	
		3rd cycle				2.70 ± 2.03	<0.001	

Values are given as mean ± SD; * values are given as a %; DBCT: double-binding randomized clinical trial; PVAS: pain visual analog scale; VAS: visual analog scale.
